# A Nursery of Typhus

**Published:** 1898-07-09

**Authors:** 


### A NURSERY OF TYPHUS.

Living in a great centre of civilisation where progress is the key-note of all social affairs, and where the very atmosphere of social life may be said to teem with philanthropy, it is well to be reminded now and again of the conditions amid which our fellow men are passing their lives, not merely in dark continents and in savage lands, but here within our own isles. In the *Caledonian Medical Journal* an account is given by Dr. Dewar of an outbreak of typhus fever in the Isle of Skye, and incidentally we learn some details of the dwellings of the crofters among whom the disease occurred. The scene of the outbreak was the township of Sconser, twelve miles distant from Portree. It is a long straggling crofting township of 38 inhabited houses, with a population of 164, one of the poorest of the Isle of Skye, and is situated by the seashore on the lower slopes of Ben Glamaig, a mountain 2,537 ft. high, which completely excludes the sunshine from the village for a considerable portion of the year. The dwelling houses are all of the usual crofter class—that is, with dry stone walls and thatched roofs, divided as a rule into a "but and ben," with a closet between, the fire-place in most cases being a hearth in the middle of the kitchen end, the sleeping room having all but invariably no fireplace. Many of the houses are built against the dug out slope of the ground, so that in some cases the land at the back is of a higher level than the roof, as a consequence of which the water from the hill oozes through the walls and sometimes makes quite a stream through the houses. The two rooms of which the cottages consist are lighted by means of small apertures (they cannot be called windows) left in the front wall, in which is fixed a piece of glass about a foot square, so that there is no ventilation except by the door. The house in which typhus fever first broke out measured 21 ft. by 11 ft. The side walls were at the front 4 ft. 9 in. in height from the floor, which was of earth, and at the back 6 ft., while the apex of the roof was only 8 ft. off the floor. There was no chimney in the sleeping room, and except two small fixed panes of glass in the front wall there was no aperture other than the door by which fresh air could be admitted. There were three beds, apparently all in the same end of the house. "The floor was sodden with wet, and littered with all manner of household *débris*, bearing no evidence of any attempt at cleanliness, and a fœtid, sickening odour pervaded the whole house. . . . The family consisted of the father, with three sons, aged respectively 19½, 16, 7, and a daughter aged 4½. The cubic space in the sleeping apartment yielded only 160 cubic feet per head. There was so little light that on my first visit, though it was but mid-day, I had to use matches to see the patients. . . . The family were in extreme poverty, and for six weeks before the outbreak of the fever they subsisted entirely on mashed potatoes and tea, of which they partook three times a day. . . . There existed with regard to the inmates of the house extreme personal uncleanness. . . . The infection in all the other cases was traced to this source. Seven other houses were infected, and the number of cases was 17 . . . I alone escaped the infection of all that visited the house." It is to be noted that more than 10 per cent. of all the dwellers in this little hamlet were laid down with typhus fever. Perhaps it is also worth noting that we as a progressive people think it our mission to spread the benefits of our religion and our civilisation (!) among the heathen, and that, according to "Burdett's Hospitals and Charities," we spend about £1,100,000 per annum in so doing!

